# Quality of life and treatment adherence in glaucoma: a cross-sectional analysis using the NEI-VFQ25 and MMAS-8 questionnaires

**DOI:** 10.5935/0004-2749.2024-0355

**Published:** 2025-09-10

**Authors:** Marina Siqueira Saito, Bernardo Kaplan Moscovici, Marcello Novoa Colombo-Barboza, Guilherme Novoa Colombo-Barboza, Luiz Roberto Colombo-Barboza, Cybelle Silva Guimarães, Priscilla Fernandes Nogueira

**Affiliations:** 1 Department of Ophthalmology, Hospital Visão Laser, Santos, SP, Brazil; 2 Departament of Ophthalmology and Visual Sciences, Escola Paulista de Medicina, Universidade Federal de São Paulo, São Paulo, SP, Brazil; 3 Centro Universitário Lusíadas, Santos, SP, Brazil; 4 Universidade Metropolitana de Santos, Santos, SP, Brazil

**Keywords:** Glaucoma, Quality of life, Patient health questionnaire, Patient acuity, Antiglaucoma agents, Visual acuity, Treat ment adherence and compliance, Surveys and questionnaires

## Abstract

**Purpose:**

To analyze the quality of life and treatment adherence of patients with glaucoma at different disease stages, considering factors such as sex, visual acuity, disease severity, and treatment characteristics.

**Methods:**

This cross-sectional study included 174 patients (346 glaucomatous eyes) recruited from clinical records and routine follow-ups at a specialized ophthalmology center. Their mean age was 39–90 years, and 60.9% of them were women. Their quality of life and adherence were assessed using the NEI-VFQ25 and MMAS-8 questionnaires, respectively. Complementary tests included 24:2 visual field test, retinography, and optical coherence tomography. Patients diagnosed with glaucoma for at least 6 months were included, whereas pregnant patients and those with ocular diseases were excluded.

**Results:**

Among the participants, 59.2% adhered to the treatment whereas 40.8% showed low adherence. The mean quality of life score was 81.87. Patients with low adherence had slightly higher quality of life scores (mean 83.1) than those with good adherence (mean 81.0), but the difference was not statistically significant. Disease severity was associated with increased optic nerve cupping, reduced thickness of the nerve fiber and ganglion cell layers, and great visual field loss. No significant correlation was observed between adherence and quality of life, indicating the independence of these factors and the influence of psychological or social elements.

**Conclusion:**

The absence of a correlation between quality of life and treatment adherence highlights the need for tailored interventions for psychological and social aspects. These findings indicate the importance of a comprehensive approach to managing glaucoma, preserving visual function, strengthening doctor–patient relationships, and considering psychosocial factors to enhance quality of life and treatment adherence.

## INTRODUCTION

Glaucoma is a leading cause of irreversible blindness worldwide, affecting approximately 76 million people; this number is projected to increase to 111.8 million by 2040^(1)^. Glaucoma is characterized by progressive optic neuropathy, leading to visual field loss and, in advanced stages, severe impairment in the quality of life (QoL)^(2^,^3)^. The burden of glaucoma extends beyond visual function loss as patients experience substantial psychosocial and economic challenges that impact their daily lives and treatment adherence^(4^,^5)^.

Studies have demonstrated that in patients with glaucoma, a higher frequency of eye drop use and increased side effects are correlated with reduced QoL, often leading to treatment discontinuation^(6)^. The chronic nature of the disease requires long-term use of intraocular pressure (IOP)-lowering medications, which may cause ocular discomfort, financial burden, and psychological stress^(7^,^8)^. Several studies have reported that medication adherence is often suboptimal, with many patients failing to comply with the prescribed regimens owing to forgetfulness, high cost, or side effects^(9^,^10)^.

The association between QoL and treatment adherence in glaucoma remains controversial. Some studies have suggested that patients with advanced disease exhibit higher adherence due to fear of vision loss, whereas others have reported no significant correlation between these factors^(11^,^12)^. QoL assessments using standardized instruments, such as the National Eye Institute Visual Function Questionnaire-25 (NEI-VFQ25), provide insights into the functional and emotional impacts of glaucoma; in contrast, self-reported scales, such as the Morisky Medication Adherence Scale-8 (MMAS-8), are often used to evaluate treatment adherence^(13^,^14)^.

The impact of sex on QoL and adherence is another critical factor, as evidence suggests that women may experience greater psychological burden and reduced QoL than men, despite similar clinical disease severities^(15^,^16)^. Additionally, disparities in access to healthcare, treatment costs, and sociocultural differences may further influence adherence patterns across different populations^(17)^.

This study aimed to analyze QoL and treatment adherence in patients with glaucoma at different stages, considering factors such as sex, visual acuity (VA), disease severity, and treatment characteristics. By investigating these associations, we aimed to identify potential areas for intervention to enhance patient outcomes and optimize glaucoma management.

## METHODS

### Study design

This cross-sectional analytical study utilized data retrospectively extracted from medical records, NEI-VFQ25 and MMAS-8 questionnaires, and complementary exams, including the 24:2 visual field test, retinography with optic nerve cupping assessment by a glaucoma specialist, and optical coherence tomography (OCT). Epidemiological data, such as age, sex, family history of glaucoma, and disease duration, were systematically collected and analyzed alongside clinical parameters, including VA, IOP, and optic nerve structural/ functional parameters. Qualitative methods were used to capture disease aspects that were challenging to evaluate through quantitative QoL measures.

Patients diagnosed with glaucoma for at least 6 months were included, whereas pregnant patients and those with other ocular diseases were excluded.

### Study population

The study was conducted at the Department of *Ophthalmology of Hospital Oftalmológico Visão Laser in Santos*, São Paulo, Brazil, from August 2022 to March 2023. It was approved by the ethics committee of *Universidade Metropolitana de Santos*, Brazil (CAAE: 64510822.3.0000.5509), and adhered to the principles of the Declaration of Helsinki. Patient confidentiality was maintained by anonymizing all data during collection and analysis.

### Inclusion and exclusion criteria

Participants aged 18 to 90 years, capable of providing informed consent, and diagnosed with glaucoma for at least 6 months were eligible for study participation. The diagnosis required evidence of optic nerve damage characterized by neuroretinal rim loss and excavation ≥0.5 in at least one eye. Patients with dementia, psychiatric or neurological disorders, other causes of visual impairment, retinal or macular diseases, optic nerve conditions unrelated to glaucoma, or a history of ocular trauma were excluded. Patients who had undergone ocular surgeries other than cataract extraction or laser procedures for glaucoma, who were pregnant, and who had immunodeficiency were also excluded from the study. These criteria were applied to ensure sample homogeneity and reduce confounding factors.

### Recruitment process

The participants were recruited through convenience sampling during routine clinical follow-ups at the study site. The inclusion criteria were confirmed by reviewing the patients’ medical records, and informed consent was obtained from them. Although convenience sampling may introduce selection bias, this approach reflects real-world clinical settings and enhances the practical relevance of the findings.

### Ophthalmological evaluation

Each participant underwent a comprehensive ophthalmological evaluation. VA was measured using a Snellen chart and converted to logMAR values for statistical analysis. IOP was measured using a Goldmann tonometer, and the mean of the last three visits was calculated for consistency. Structural/functional assessments included the 24:2 visual field test (Humphrey Field Analyzer II 740i^®^, Zeiss, Oberkochen, Germany), retinography (Visucam 500^®^, Oberkochen, Germany), and OCT (Heidelberg Spectralis^®^, Heidelberg, Germany). Corneal pachymetry was performed to assess corneal thickness, as thinner corneas may be associated with a more advanced disease. For the corneal evaluation, an anterior segment OCT system was used. These exams were conducted by trained nursing technicians under the supervision of a glaucoma specialist.

### Questionnaires and validation

The NEI-VFQ25 and MMAS-8 questionnaires were administered during the patients’ scheduled follow-up visits, ensuring uniformity in timing and minimizing recall bias. The visits occurred between August 2022 and March 2023. A trained ophthalmologist conducted the survey in a private and controlled environment to ensure consistency and accuracy. On average, the questionnaires took 15 to 20 min to complete. Both questionnaires were administered as individual interviews rather than self-administered to account for potential patient comprehension or literacy variability.

The Portuguese versions of the NEI-VFQ25 and MMAS-8 questionnaires were used, which have both been culturally adapted for the Brazilian population. Although the MMAS-8 has not been specifically validated for glaucoma in Brazil, its previous application in ophthalmology studies supports its relevance. The NEI-VFQ25 scores were calculated as a simple average of responses across subdomains, excluding “general health”, with scores ranging from 0 to 100, where higher scores indicated better QoL. Meanwhile, the MMAS-8 scores ranged from 0 to 8, with ≥6.0 indicating adherence and scores between 0 and 5.75 indicating nonadherence.

### Questionnaire administration and scoring

A trained ophthalmologist administered the NEI-VFQ25 and MMAS-8 questionnaires to ensure consistency and reliability. NEI-VFQ25 is a 25-item tool designed to assess QoL across three domains: general health and vision, difficulty with activities, and responses to vision problems. The scores range from 0 to 100, with higher scores indicating better QoL. In contrast, MMAS-8 is an 8-item scale used to evaluate treatment adherence. The scores range from 0 to 8, with a score ≥6 indicating adherence and scores between 0 and 5.75 indicating nonadherence. The questionnaires were administered in a private setting during scheduled clinical visits, with an average completion time of 15 to 20 min.

### Sample-size calculation

A pilot study involving 20 glaucomatous eyes was conducted to evaluate the feasibility of the study instruments, identify challenges in data collection, and estimate the variability in NEI-VFQ25 scores. The sample size was calculated using the standard deviation (σ) observed in the pilot study.

The calculation determined that 294 glaucomatous eyes were required. To account for potential dropouts or incomplete data, 10% was added, resulting in a final sample size of 324 glaucomatous eyes. This sample size ensured adequate statistical power for subgroup analyses and robust evaluations of the correlations between clinical variables, QoL, and adherence.

### Statistical analysis

All data were analyzed using SPSS Statistics, version 27 (IBM Corp., Armonk, NY). Continuous variables were expressed using means and standard deviations, whereas categorical variables were expressed as frequencies and percentages. Multivariate regression models were used to evaluate the associations between QoL, adherence, and clinical parameters, controlling for potential confounders. The two-proportion Z-test was employed to compare the proportions of qualitative factors, and Spearman’s correlation was used to evaluate the associations between the questionnaire scores and the quantitative clinical variables. The Kruskal–Wallis test was used to assess differences in adherence levels relative to the QoL scores. The assumptions of normality, homogeneity of variance, and independence were tested before applying statistical methods.

### Limitations and bias mitigation

Potential limitations included recall bias associated with self-reported questionnaires and the inability of cross-sectional designs to establish causality. To minimize inconsistencies, standardized protocols for data collection and questionnaire administration were implemented. Despite these limitations, the methodology provides a robust framework for understanding the associations between clinical parameters, QoL, and adherence in patients with glaucoma.

## RESULTS

### Sample characteristics

The study included 174 patients (346 glaucomatous eyes) with a mean age of 65.2 ± 11.4 (range, 39–90) years. Of the patients, 60.9% were women (χ^2^, p<0.001) and 70.5% reported a positive family history of glaucoma (χ^2^, p<0.001). Two eyes were excluded due to insufficient follow-up data and poor-quality imaging, thereby meeting the predefined exclusion criteria of the study (Table 1).

**Table 1:** Descriptive statistics of quantitative factors (346 eyes)

Descriptive statistics of the clinical parameters	Mean ± SD	Range	Units
Age	65.2 ± 11.4	39.0–90.0	Years
Visual acuity (VA)	0.06 ± 0.04	0.00 - 1.30	logMAR
Intraocular pressure (IOP)	16.8 ± 3.2	10.0–32.0	mmHg
Pachymetry	525.7 ± 36.2	440.0–610.0	µm
Retinal nerve fiber layer	82.3 ± 12.1	50.0–110.0	µm

SD = standard deviation. All abbreviations are defined.

### Pachymetry

The mean central corneal thickness was 525.7 ± 36.2 (range, 440–610) µm. Patients with thinner corneas (<500 µm) had significantly lower QoL scores (mean NEI-VFQ25: 76.2 ± 10.5, p=0.009) than those with normal corneas (mean NEI-VFQ25: 83.7 ± 11.3). The disease stage and extent of visual field loss were defined according to the mean deviation (MD) and visual field index (VFI) values, as detailed in the Methods section (Table 1).

### Eye drop usage

Beta-blockers were the most frequently prescribed medication (77.5%, χ^2^, p<0.001), followed by prostaglandin analogs (62.1%) and carbonic anhydrase inhibitors (48.3%). Combined eye drop therapy (≥2 medications) was used by 48.6% of the patients, whereas single-therapy regimens were employed by 51.4%. The number of participants (n) was added in parentheses following the percentages: 77.5% (n=135), 62.1% (n=108), 48.3% (n=84), 48.6% (n=85), and 51.4% (n=89). Combined therapy was associated with lower QoL scores (mean NEI-VFQ25: 78.2 ± 10.7, p=0.022) compared with single therapy (mean NEI-VFQ25: 83.1 ± 11.9, p=0.022) (Table 2).

**Table 2:** Distribution of eye-drop usage

Type of medication	Patients (%)	p-value
Single eye drop	51.4%	p<0.001
Combined therapy	48.6%	p<0.001
Beta-blockers	77.5%	p<0.001
NEI-VFQ25 Score (Mean ± SD)	83.1 ± 11.9	78.2 ± 10.7

### Visual acuity

The mean VA across all eyes was 0.06 ± 0.04 (range, 0.00–1.30) logMAR. For qualitative assessments, standardized logMAR values were assigned: “Hand Movement” as 2.3, “Light Perception” as 2.7, and “No Light Perception” as 3.0. A significant negative correlation was found between the VA and QoL scores (NEI-VFQ25) (r=−0.278, p<0.001), suggesting that better visual function was strongly associated with higher QoL. These findings are detailed in table 3.

**Table 3:** Correlations between clinical variables and quality of life/treatment adherence

Parameter	Correlation (r)	p-value
Visual acuity (VA)	-0.278	p<0.001
RNFL thickness	0.271–0.351	p<0.001
Visual field index (VFI)	-0.312	p<0.001

RNFL= Retinal nerve fiber layer.

### Intraocular pressure

The mean IOP was 16.8 ± 3.2 (range, 10–32) mmHg. Normal IOP (10–21 mmHg) was observed in 87.6% of the eyes, whereas elevated IOP (≥21 mmHg) was recorded in 12.4%. The mean NEI-VFQ25 scores for the normal IOP and elevated IOP groups were 82.5 ± 10.9 and 78.5 ± 11.2, respectively (p=0.014) (Table 3).

### Retinal nerve fiber layer

The mean RNFL thickness was 82.3 ± 12.1 (range, 50–110) µm. A significant positive correlation was observed between RNFL thickness and QoL scores (r value ranging from 0.271 to 0.351 for different quadrants, all p<0.001). The mean NEI-VFQ25 scores for the normal RNFL thickness and thinner RNFL groups were 84.0 ± 9.8 and 75.6 ± 12.7, respectively (p<0.001).

### Visual field analysis

The mean VFI was 68.5% ± 21.4% (range, 20%–100%), whereas the MD was −6.8 ± 5.1 (range, −26.0–0.0) dB. Visual field defects were categorized into moderate-to-severe (MD <−6 dB) and normal-to-mild (MD ≥−6 dB), as detailed in the Methods section. Moderate-to-severe visual field defects were observed in 54% of the eyes and were significantly associated with reduced QoL (mean NEI-VFQ25 scores: 73.8 ± 12.9 for moderate-to-severe defects vs. 85.2 ± 10.3 for normal-to-mild defects, p<0.001) (Table 3).

### QoL and adherence

The mean NEI-VFQ25 score was 81.87 ± 12.3 (range, 40–100). Patients in the advanced stage of the disease had significantly lower QoL scores (mean NEI-VFQ25: 71.6 ± 14.2, p<0.001) than those in the nonadvanced stage (mean NEI-VFQ25: 86.9 ± 9.5). The adherence groups also significantly differed in QoL, with mean NEI-VFQ25 scores of 77.9 ± 13.4 for the low adherence group and 84.2 ± 11.1 for the good adherence group (p=0.017) (Tables 3 and 4).

**Table 4:** Comparative analysis between the low and good adherence groups

Parameter	Low adherence (Mean ± SD)	Good adherence (Mean ± SD)	p-value
Visual acuity (VA)	0.08 ± 0.05	0.05 ± 0.03	0.015
Treatment time (months)	45.6 ± 12.4	38.2 ± 10.7	0.022
Visual field index (VFI)	65.3 ± 18.9	72.4 ± 19.8	0.008
MD **(dB)**	-7.5 ± 3.2	-6.0 ± 2.8	0.013
Pattern Std. Dev. (**PSD)**	3.2 ± 0.7	2.8 ± 0.6	0.020
Pachymetry (µm)	512.3 ± 30.2	525.7 ± 28.3	0.018
NEI-VFQ25 Score	77.9 ± 13.4	84.2 ± 11.1	0.017

PSD= Pattern Standard Deviation.

## DISCUSSION

This study reinforces the World Health Organization’s definition of health, which considers the absence of disease alongside the individual’s perception of physical, mental, and social well-being. Glaucoma signifi cantly affects patients’ QoL owing to various factors, including visual function loss, challenges in daily treatment routines, medication side effects, treatment costs, and psychological burden of living with a chronic, sight-threatening disease. These factors were evident in our findings, particularly among patients with poor VA who had lower QoL scores^(18^-^20)^.

Visual field loss, a hallmark of glaucoma progression, significantly affects patients’ QoL by reducing functional independence. Our results suggested that patients with poor VA also had significantly compromised visual fields, indicated by lower VFI and higher MD values. These findings highlight the critical role of the peripheral vision in daily activities and overall QoL. Notably, Abe et al. reported that eyes with less severe glaucoma at baseline are more likely to demonstrate progression through structural assessments, such as spectral-domain OCT. In contrast, more advanced eyes predominantly progress through functional changes detected by standard automated perimetry. This highlights the stage-dependent nature of disease progression and reinforces the importance of tailored monitoring strategies^(1^,^18^-^21)^.

Our results indicated that patients with better corrected VA had higher QoL scores, enabling them to more effectively maintain their daily activities. However, as Guimarães et al. reported, even patients with good VA may experience difficulty reading, emphasizing that QoL can be compromised despite apparently normal VA. Machado et al. expanded on this finding, showing that patients with advanced glaucoma (MD <−12 dB) have significantly lower QoL scores, particularly when visual function in the worse eye is severely compromised. These findings indicate the importance of assessing functional limitations beyond VA to comprehensively address patients’ needs^(21^-^29)^.

Complementary tests, such as the 24:2 visual field test and OCT, provided structural/functional insights into disease progression. Patients with better preserved visual fields (higher VFI and lower MD and pattern standard deviation) had higher QoL scores, albeit lower treatment adherence. This contrasts with patients exhibiting more advanced alterations, such as thinner RNFL and greater optic nerve cupping, who demonstrated poorer QoL but higher adherence. Gracitelli et al. reported that each 1 µm/year loss in RNFL thickness corresponds to a 1.3-unit decrease in NEI-VFQ25 scores, reinforcing the role of RNFL as a critical biomarker for assessing the impact of glaucoma on patient well-being. In addition, clinical guidelines highlight the need to integrate structural/functional assessments to effectively monitor disease progression, as highlighted in evidence-based approaches to lowering IOP through medical, laser, or surgical interventions. Early detection and targeted treatment strategies remain pivotal in the preservation of visual function and improvement of QoL in patients with glaucoma^(1^,^18^-^29)^.

Our findings align with those of Lee et al. who reported that patients with mild visual field defects maintain higher QoL than those with moderate-to-severe field loss. Early detection and intervention are necessary to preserve visual fields and enhance QoL in patients with glaucoma. Furthermore, Machado et al. emphasized the centrality of visual field preservation, particularly in the better-seeing eye, to maintaining patient independence and reducing functional limitations^(5^,^18^-^29)^.

Patients with thinner corneas had worse QoL scores and more advanced visual field defects, supporting previous findings indicating that corneal biomechanics may influence glaucoma severity. These findings highlight the importance of corneal parameters in disease monitoring^(6^,^27^,^29)^.

The treatment duration was inversely associated with QoL, reflecting the cumulative burden of years of daily eye drop use. Mangione et al. found that QoL often declines at treatment initiation but improves over time as patients adjust. However, our findings indicate that some patients experience persistent negative effects, highlighting the need for individualized support throughout the treatment. Financial constraints and psychological stress may compound these effects, particularly in resource-limited settings^(14)^.

As regards adherence, patients with longer treatment durations exhibited greater compliance, likely due to increased disease awareness and familiarity with medication use. Similarly, a positive family history was associated with higher adherence. Torrance et al. corroborated these findings, noting that patients in advanced disease stages demonstrate higher treatment adherence, driven by the fear of further vision loss. However, these patients also face great challenges in medication administration, particularly those with advanced optic nerve damage and lower VA^(21)^.

The association between perceived QoL and treatment adherence is further supported by several studies, such as those by Cho et al. This study assessed parti cipants in the Support, Educate, Empower program and found that better perceived function in near-vision activities was significantly associated with higher adherence to glaucoma treatment. Specifically, a 10-unit increase in perceived near-vision function resulted in a 2.2% increase in medication adherence, even after adjustment for confounding factors, such as age, gender, race, and income. These findings suggest that interventions to improve vision-related QoL, particularly in near-vision activities, could directly enhance treatment adherence in patients with glaucoma. This highlights the importance of addressing the functional and emotional aspects of their daily lives as part of a comprehensive disease management plan^(19)^.

Unexpectedly, patients using combined eye drop therapy did not show significantly higher adherence, despite the assumption that fewer medications improve compliance. Of the 64 patients using combined therapies, 21.1% demonstrated low adherence. However, as shown in table 2, combined therapy was used by 48.6% of the total participants (n=174), rather than the stated 64. This discrepancy has been corrected**.** Financial burden remains a significant barrier, even when the regimens are simplified. Policies to subsidize combination therapies could mitigate these barriers and improve the treatment outcomes^(8^,^21)^.

While this study provides valuable insights, certain limitations warrant acknowledgment. The cross-sectional design prevents the establishment of causality, and the MMAS-8 scale has not been specifically validated for patients with glaucoma in Brazil. In addition, the relatively small sample-size limits generalizability to clinical practice. Future studies should address these limitations through longitudinal designs, larger cohorts, and validation of adherence tools for this population.

The integration of structural/functional assessments, such as RNFL and visual field measurements, into routine care could optimize monitoring and patient outcomes. Moreover, targeted interventions, including patient education programs, financial support, and psychological counseling, are critical for the enhancement of adherence and QoL. Gracitelli et al.’s findings on RNFL loss and QoL provide a compelling case for incorporating structural biomarkers into patient management^(1^,^21^,^29)^.

In conclusion, this study demonstrates that glaucoma significantly impacts QoL and treatment adherence. Patients with better VA and less severe disease had higher QoL, whereas those with advanced disease demonstrated greater adherence, probably driven by fear of progression**.** These findings highlight the importance of early intervention, patient education, and systemic support to mitigate the burden of glaucoma. By prioritizing a holistic approach to disease management, healthcare providers can enhance patient outcomes and improve their overall QoL.

## Data Availability

The datasets generated during and/or analyzed during the current study are available on demand from referees.
